# *Camphora officinarum* (Syn. *Cinnamomum camphora*): Botany, Phytochemistry, Biological Activities, Agro-Industrial Applications, and Biotechnology

**DOI:** 10.3390/plants15101467

**Published:** 2026-05-12

**Authors:** Kamran Shah, Wenjun Dai, Qinyuan Shen, Yanjun Zhang, Junhan Guo, Jiashuang Qiao, Jiaxin Hu, Liangye Huang, Daoliang Yan, Yongjun Wang, Jianfang Zuo, Yuanyuan Li, Huwei Yuan, Bingsong Zheng

**Affiliations:** 1 State Key Laboratory for Development and Utilization of Forest Food Resources, Zhejiang A&F University, Hangzhou 311300, China; kamranshah801@zafu.edu.cn (K.S.); 2023602122015@stu.zafu.edu.cn (W.D.); 2024202011016@stu.zafu.edu.cn (Q.S.); bamboocenteroffice@163.com (Y.Z.); guojh@stu.zafu.edu.cn (J.G.); 2025602122079@stu.zafu.edu.cn (J.Q.); hujiaxin123@stu.zafu.edu.cn (J.H.); 2023602121036@stu.zafu.edu.cn (L.H.); liangsie@zafu.edu.cn (D.Y.); wangyj@zafu.edu.cn (Y.W.); zuojianfang@zafu.edu.cn (J.Z.); lyy@zafu.edu.cn (Y.L.); 2 Zhejiang Key Laboratory of Non-Wood Forest Products Quality Regulation and Processing Utilization, Zhejiang A&F University, Hangzhou 311300, China; 3 College of Horticulture, South China Agricultural University, Guangzhou 510642, China; 4 College of Horticulture, Northwest A&F University, Yangling 712100, China; 5 China National Bamboo Research Center, Hangzhou 310012, China

**Keywords:** *Camphora officinarum*, *Camphor* tree, terpenoids, essential oils, biotechnology

## Abstract

*Camphora officinarum* (syn. *Cinnamomum camphora*) is an ecologically, medicinally, and economically important tree species widely known for its essential oils (EOs), timber, and long history of use in traditional medicine. In recent years, renewed interest in this species has been driven by taxonomic revision, the discovery of chemically distinct chemotypes, and advances in genomics, metabolomics, and biotechnological processing. This review summarizes current knowledge on the botany, distribution, phytochemistry, biological properties, agro-industrial value, and biotechnological potential of *C. officinarum*. Particular attention is given to the genetic and metabolic basis of terpene diversity, especially the role of terpene synthase (TPS) gene expansion in the formation of camphor-, linalool-, borneol-, cineole-, and citral-type profiles. We also discuss developments in essential oil extraction, the utilization of non-volatile constituents such as flavonoids and lignans, and the nutritional value of seed kernel oil rich in medium-chain fatty acids (MCFAs). In addition, recent progress in tissue culture, multi-omics analysis, metabolic engineering, and nano-enabled delivery systems is reviewed. The paper also considers important safety and ecological issues, including the dose-dependent toxicity of camphor and the contrasting status of the species as a protected native resource in East Asia and an invasive plant in some introduced regions. Overall, this review provides an updated and balanced overview of *C. officinarum*, identifies key knowledge gaps, and highlights future prospects for sustainable utilization, conservation of native genetic resources, and exploitative control of invasive populations.

## 1. Introduction

### 1.1. Botanical Taxonomy and Nomenclature

The camphor tree has long been known in the literature as *Cinnamomum camphora* (L.) J. Presl. However, recent phylogenetic studies have led to a revision of its placement within Lauraceae. Molecular analyses based on multiple loci have shown that the traditional broad circumscription of *Cinnamomum* included lineages that do not form a single natural group. In particular, species formerly categorized under *Cinnamomum* sect. *Camphora* form a distinct clade that is more closely related to *Sassafras* than to *Cinnamomum sensu stricto* [1]. This phylogenetic pattern is also supported by leaf epidermal micromorphology, which helps distinguish the *Camphora* lineage from other related groups [2]. On this basis, the genus *Camphora* Fabr. has been reinstated, and the accepted name of the camphor tree has been treated as *C. officinarum* Nees in recent taxonomic work [3].

Although this taxonomic change improves the alignment between classification and evolutionary relationships, nomenclature in the recent literature is still mixed. Many ecological, phytochemical, horticultural, and pharmacological studies continue to use the name *C. camphora*, whereas newer taxonomic and phylogenomic studies increasingly adopt *C. officinarum*. For this reason, both names remain important when surveying the literature. In the present review, we use *C. officinarum* as the principal name and note *C. camphora* as its widely used synonym where relevant.

The evolutionary history of this lineage also remains an active area of research. Recent comparisons of plastome and nuclear ribosomal DNA datasets suggest that relationships within Cinnamomeae are not yet fully resolved. Nuclear data generally support the monophyly of *Camphora*, whereas plastome-based analyses have produced less consistent topologies [4]. In addition, plastid phylogenomic studies have revealed substantial variation among East Asian populations, including structural differences in chloroplast genomes [5]. These patterns may reflect incomplete lineage sorting, historical hybridization, or chloroplast capture. Therefore, while the recognition of *C. officinarum* provides a clearer taxonomic framework, broader sampling and integrated genomic analyses are still needed to clarify the evolutionary history of the group.

### 1.2. Origin, Geographical Distribution, and Habitat

*C. officinarum* is native to East Asia and is mainly distributed in southern China, Taiwan, Japan, and the Korean Peninsula [6,7]. However, a significant biogeographic discrepancy exists regarding the species’ status in mainland China. While the *Flora of China* records the camphor tree as a native component of subtropical evergreen broad-leaved forests [6], the Plants of the World Online (POWO) database, a recognized global authority, categorizes mainland China, including the southern provinces, as a region of introduction [7]. According to the POWO database, the species is recognized as native only to Japan, Korea, and Taiwan [7]. This discrepancy is of fundamental importance, as it influences the assessment of the species’ conservation status and the interpretation of its genetic resources. If the populations in mainland China are the result of historical introduction, the high levels of genetic and chemotypic diversity found in the region likely reflect centuries of human-mediated translocation and selective cultivation rather than purely indigenous evolution. Acknowledging this status is critical for accurately framing conservation strategies and evaluating the species’ invasive potential in other introduced ranges. In its native range, it is an important component of subtropical evergreen broad-leaved forests and is often found in warm, humid environments with sufficient rainfall [8]. Because of its ecological adaptability and long cultivation history, the species now occurs across a wide range of natural, semi-natural, and managed landscapes.

Recent phylogeographic and plastid genomic studies suggest that wild and cultivated populations of *C. officinarum* are more structured than previously assumed. Populations from different parts of East Asia show clear geographic differentiation, indicating that regional lineages have been shaped by both environmental heterogeneity and historical isolation [5]. At the same time, some populations show evidence of admixture, which is likely related to long-term human movement of planting materials, ornamental use, and forestry cultivation. As a result, the present distribution of the species reflects both natural biogeographic history and prolonged anthropogenic influence.

Within its native area, *C. officinarum* also has considerable conservation value. Old and large trees preserved in urban, rural, temple, and forest landscapes represent important reservoirs of genetic diversity and local adaptation [9]. These long-lived individuals may be particularly valuable for future conservation and breeding programs, especially where wild genetic resources have been reduced by land-use change or replacement by planted material.

Outside East Asia, *C. officinarum* has been widely introduced for ornamental planting, timber production, and EO use. In several subtropical and tropical regions, the species has become naturalized and, in some cases, invasive. Reports from eastern Australia and the southeastern United States show that it can spread beyond cultivation, form dense stands, and compete strongly with native vegetation [10]. This contrast gives the species a dual ecological identity: it is a native genetic resource that deserves conservation in part of its range but a management concern in some introduced environments.

Climate change adds another dimension to the future distribution of *C. officinarum*. Modeling studies based on maximum entropy (MaxEnt) have suggested that suitable habitats in China may shift under future climate scenarios [11]. More recent work further indicates that climate change may influence the geographic pattern of major essential-oil chemotypes, including linalool-, borneol-, and citral-dominant forms [12]. Because these chemotypes are of both ecological and industrial interest, changes in habitat suitability may affect not only species distribution but also the regional availability of valuable chemical traits. For this reason, future conservation, germplasm management, and plantation planning should consider both species-level distribution and chemotype-specific responses to climate.

### 1.3. Historical and Economic Significance

*C. officinarum* has long held cultural, medicinal, and commercial importance. Historically, natural camphor obtained from this tree was a valued commodity in regional and intercontinental trade, and it was widely used in perfumery, ritual practice, preservation, and traditional medicine [13]. In East Asia and other traditional medical systems, camphor and related plant materials were used for a range of purposes, including the relief of pain, inflammation, respiratory discomfort, and infectious conditions [14,15]. In addition to its medicinal value, the species has also been appreciated for its timber, fragrance, shade, and ornamental character, which contributed to its long history of cultivation in both rural and urban landscapes [16].

Its economic importance continues today, although the main focus of utilization has shifted. Earlier use was often centered on timber and the general extraction of camphor from wood and other tissues. More recently, attention has moved toward the selective use of leaves, seeds, and specialized chemotypes for EOs, functional lipids, phytochemicals, and biotechnological applications. This shift reflects a broader move from bulk exploitation toward more targeted and value-added use of specific plant fractions.

One of the most important features supporting this transition is the marked chemical diversity found within the species. Different chemotypes produce distinct dominant terpenoids, such as linalool, borneol, camphor, cineole, and citral, and these profiles are directly relevant to fragrance, flavor, medicinal, and industrial markets. As a result, interest in *C. officinarum* is no longer limited to traditional camphor production. Instead, the species is increasingly viewed as a source of diverse natural products with differentiated commercial uses. For example, linalool-rich materials are of interest to the fragrance and cosmetic sectors, whereas borneol- and citral-related products have attracted attention in pharmaceutical and fine chemical research. Furthermore, camphor- and cineole-rich extracts remain highly valued in the pharmaceutical and flavor industries, particularly as active ingredients in topical analgesics, respiratory treatments, and antimicrobial formulations [17]. Recent specialized studies and comprehensive reviews have extensively documented the commercial importance of these five major chemotypes, detailing their multifaceted applications across the global fragrance, flavor, cosmetic, and pharmaceutical sectors [17,18,19].

At the same time, advances in genomics, transcriptomics, and metabolomics are changing how the species is studied and utilized. Recent chromosome-level genome assemblies and functional studies on terpene biosynthesis have improved our understanding of the molecular basis of chemotype formation and metabolic regulation [20,21,22]. This has practical implications for germplasm evaluation, cultivar development, and the more efficient production of target compounds. In this sense, *C. officinarum* is becoming important not only as a traditional forest resource but also as a model for linking plant secondary metabolism with modern breeding and biotechnological development.

Despite this growing industrial relevance, sustainable use remains essential. Continued commercial development should be balanced with the conservation of wild and old-tree genetic resources, especially in native regions where unique lineages and locally adapted chemotypes may be underrepresented in plantation systems. A major challenge for future research and industry will therefore be to integrate historical knowledge, modern molecular tools, and sustainable resource management in a way that supports both economic use and long-term conservation.

The primary objective of this review is to provide an updated and balanced synthesis of current knowledge on *C. officinarum*, covering its taxonomy, botany, phytochemistry, biological activities, agro-industrial applications, biotechnology, safety issues, and ecological challenges. Rather than presenting the species only as a source of valuable natural products, the review also considers the knowledge gaps that currently limit its wider application, including the need for standardized chemotype evaluation, stronger toxicological and clinical evidence, improved biotechnological production systems, and region-specific ecological management. Particular attention is given to future prospects for sustainable utilization, including integrated biorefinery strategies, precision breeding, microbial biomanufacturing, and the potential use of “harvest-for-control” approaches to exploit invasive populations while supporting ecological restoration.

### 1.4. Literature Search Methodology and Bibliometric Analysis

To ensure a comprehensive and contemporary synthesis, a systematic literature search was conducted across major academic databases, including Web of Science, PubMed, Scopus, and Google Scholar. The search strategy utilized primary keywords such as “*Camphora officinarum*,” “*Cinnamomum camphora*,” “essential oils,” “chemotypes,” and “biotechnology,” with a specific focus on high-impact studies published between 2018 and 2025. Bibliometric trends indicate a significant exponential increase in research output related to the “Camphora” lineage following its recent taxonomic reinstatement and the publication of chromosome-level genome assemblies. Emerging research clusters are increasingly focused on multi-omics integration, synthetic biology (e.g., microbial metabolic engineering), and advanced nano-enabled delivery systems, reflecting a global shift from traditional forestry toward precision biomanufacturing. This review prioritizes peer-reviewed articles that bridge the gap between historical ethnomedicinal use and modern mechanistic validation.

## 2. Plant Biology and Ecology

### 2.1. Morphology and Anatomy

**Figure 1:**
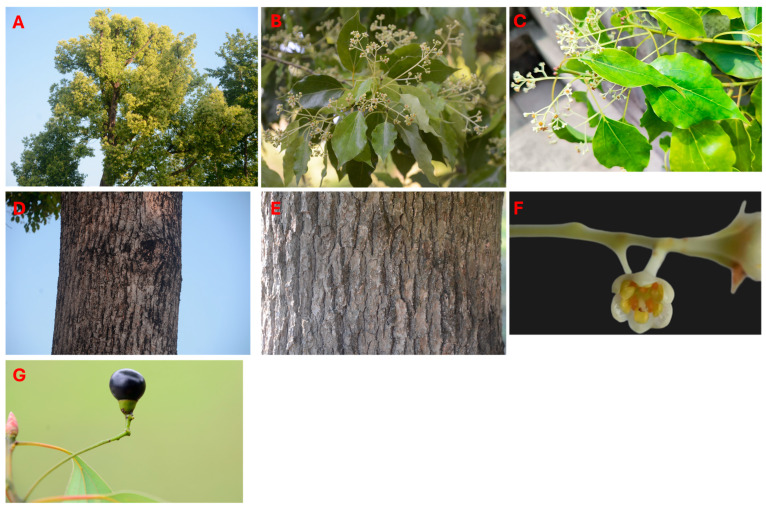
Morphological phenotypes of the camphor tree (*C. officinarum*): (**A**) crown; (**B**,**C**) leaves and inflorescences; (**D**,**E**) bark; (**F**) flower; (**G**) fruit.

The camphor tree, *C. officinarum*, has long been described and identified through a set of easily recognizable morphological characters: evergreen crowns, alternate and pinnately veined leaves with conspicuous domatia in the vein axils, and thick, fissured bark on older trunks (Figure 1). These macroscopic traits are still useful taxonomically, but recent work in Cinnamomum, particularly regarding secretory oil cells and leaf morphology, has shifted attention toward how anatomical structures relate to chemical profiles and ecological performance [23,24,25]. In particular, studies of leaf development show that the formation, size, and density of secretory oil cells are key determinants of when and where EOs accumulate within the leaf. In citral-dominant chemotypes, the rapid expansion of these oil cells around the time of leaf maturation provides a practical anatomical indicator for deciding optimal harvest dates and for improving the yield and quality of citral-rich oils [23]. Comparative analyses across provenances further indicate that differences in leaf area, stomatal characteristics, and epidermal micromorphology are closely associated with specific chemotypes and geographic origins and that these traits influence seasonal patterns of photosynthesis and resource use [2,24,25].

Anatomical studies of stems and roots have revealed a similar level of functional complexity. Although commercial essential-oil production has traditionally focused on leaves, developmental transcriptomic and metabolomic data indicate that young stems function as active metabolic sinks. During secondary growth, the deposition of secondary cell walls is coordinated with increased terpenoid biosynthesis, suggesting that mechanical strengthening and chemical defense are tightly coupled processes in developing shoots [26]. Below ground, the root system shows marked structural plasticity. High-resolution observations using rhizotrons demonstrate that *C. officinarum* responds to mechanical disturbance, such as trunk leaning, by reallocating root biomass asymmetrically and adjusting root angles. These adjustments improve anchorage and allow the tree to continue exploiting heterogeneous soil patches for water and nutrients [27].

Recent experimental work has also started to manipulate anatomy directly as a breeding target. Colchicine-induced chromosome doubling has been used to generate polyploid lines of *C. officinarum* (reported under *C. camphora* in earlier studies). Polyploid plants show enlarged leaves and other macroscopic changes, but importantly, they also develop larger and more abundant storage structures for terpenoids [28]. These alterations suggest that genome doubling can be used to jointly modify plant form and internal secretory tissues, providing a promising route to cultivars with higher essential-oil productivity. Taken together, current research moves beyond purely descriptive morphology and treats anatomical traits as integrative markers that link genetic background, chemical composition, and ecological performance, as well as actionable targets for crop improvement.

### 2.2. Ecophysiology and Growth Dynamics

The growth and survival of *C. officinarum* are underpinned by a flexible ecophysiological strategy that links canopy structure, photosynthetic regulation, and secondary metabolism. In mature trees, carbon gain is strongly influenced by architectural traits such as leaf orientation, vertical position in the crown, and associated variations in light interception and mesophyll conductance [29]. These structural factors define the baseline photosynthetic capacity under favorable conditions. At the same time, the species shows a broad tolerance to environmental stress, adjusting both primary and secondary metabolism to maintain functioning under heat, drought, and poor soil conditions.

A key element of this plasticity is the active role of volatile organic compounds (VOCs), especially monoterpenes such as camphor, linalool, and 1,8-cineole. Rather than acting only as defensive compounds or commercial products, these volatiles also participate directly in stress physiology. Under acute high-temperature episodes, trees rapidly increase monoterpene emissions, which contributes to the scavenging of reactive oxygen species (ROS) and helps protect the photosynthetic machinery from heat-induced oxidative damage [30]. Experimental work on drought responses has highlighted a similar coupling between environmental stress and terpene production. Under mild-to-moderate water deficit, carbon allocation shifts toward terpenoid biosynthesis, with a marked increase in the accumulation of citral and related compounds, while carefully managed light and water regimes can still support acceptable biomass production [31]. These findings point to a practical trade-off: moderate, controlled stress can be used as an elicitor to enhance essential-oil yield, whereas more severe or prolonged stress leads to irreversible damage and growth loss.

*C. officinarum* also performs well in urban and degraded habitats. In paved environments, trees coordinate leaf hydraulic traits with economic traits (such as specific leaf area and nitrogen content) to maintain hydration and photosynthetic activity despite restricted soil volumes and altered microclimates [32]. Below ground, the species shows notable tolerance to heavy metal contamination. Under excessive copper stress, for example, roots reorganize their architecture and adjust antioxidant activity in a spatially heterogeneous manner, confining oxidative damage and metal accumulation to specific root regions and thereby limiting impacts on nutrient balance and shoot photosynthesis [33,34]. These responses make *C. officinarum* a promising candidate for ecological restoration on contaminated or disturbed sites. Overall, the available evidence portrays the species as a physiologically agile tree that can adjust growth, carbon use, and secondary metabolism to cope with, and sometimes capitalize on, environmental stress.

### 2.3. Chemotypes and Genetic Diversity

The most distinguishing biological hallmark of *C. officinarum* is its profound “chemical polymorphism”, which refers to the occurrence of morphologically indistinguishable trees that synthesize completely divergent dominant EOs. Historically classified into camphor, linalool, cineole, borneol, and nerolidol types, the recent discovery and commercialization of the rare, highly lucrative citral chemotype have revitalized interest in the species’ metabolic mapping [22,23]. Advanced multi-omics integrations have decisively moved the field from merely cataloging these volatile profiles to unraveling their strict genomic regulation. Recent chromosome-level genome assemblies reveal that the immense diversity of terpenoids in *C. officinarum* was evolutionarily catalyzed by two ancestral whole-genome duplication (WGD) events. These events triggered the massive tandem duplication and functional divergence of the TPS gene superfamily [20,35]. Further transcriptomic mining has identified specialized master-switch genes, such as geraniol synthase (*CoGES*) and specific alcohol dehydrogenases, that dictate the precise metabolic bifurcation of distinct monoterpenoids [21].

Beneath this chemical complexity lies a deeply structured yet highly fluid genetic diversity that has been historically underestimated. The application of high-resolution plastid phylogenomics, genome-wide single-nucleotide polymorphism discovery, and EST-SSR markers has recently decoded the complex phylogeography of the species across East Asian subtropical forests [36,37]. While certain regions display distinct evolutionary lineages characterized by genomic structural expansions, such as lineage-specific elongations of inverted repeats within the chloroplast genome, the broader population structure reflects extensive admixture [5]. This genomic mosaic is the direct consequence of millennia of human-mediated translocation, intensive historic cultivation, and natural introgressive hybridization with closely related species, such as the endangered *Cinnamomum kanehirae* [38]. Consequently, genetic markers are now indispensable for untangling traditional taxonomic misclassifications and accurately identifying elite germplasm.

**Figure 2:**
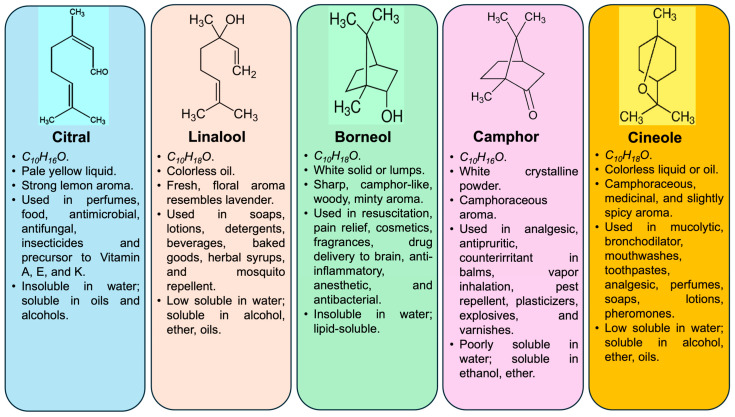
The chemotype structure, appearance, aroma, uses, and solubility of major essential oils of the camphor tree (*C. officinarum*).

Understanding the nexus between genetic architecture and chemical polymorphism is becoming increasingly urgent in the face of global climate change. Predictive habitat modeling utilizing MaxEnt algorithms suggests that shifting climatic variables, particularly mean annual precipitation, will precipitate a severe spatial redistribution of suitable habitats for specific EOs chemotypes across Southern China [12]. Because these chemotypes are highly sensitive to their specific agro-climatic niches, this impending geographic shift threatens wild populations and regional supply chains. Therefore, contemporary research emphasizes the critical necessity of prioritizing chemotype-specific, marker-assisted conservation strategies and the accelerated development of climate-resilient cultivars, such as the newly registered ‘Jilong 1’ [39], to secure the future of the *Camphora* bioeconomy. A detailed overview of the major essential oil chemotypes, their key biosynthetic drivers, and industrial applications is summarized in Table 1 and Figure 2.

## 3. Phytochemistry and Nutritional Profile

### 3.1. Essential Oils and Volatile Compounds

The commercial and pharmacological valorization of *C. officinarum* has historically been driven by its profound intraspecific chemical polymorphism, which yields EOs dominated by distinct monoterpene profiles. Depending on the prevalent volatiles, most notably camphor, linalool, eucalyptol (1,8-cineole), borneol, or the highly sought-after citral, populations are categorized into specialized chemotypes. Rather than relying on mere phytochemical cataloging, contemporary multi-omics research integrates transcriptomics and metabolomics to elucidate the dynamic spatiotemporal regulation of terpene biosynthesis. For instance, recent functional characterizations of dedicated geraniol synthases and transcription factors have decoded the molecular architecture that dictates the hyperaccumulation of specific compounds, such as citral, during early leaf and stem development [21,22]. This paradigm shift from descriptive botany to functional genomics provides a crucial biotechnological roadmap for precision cultivation, enabling the targeted elicitation of high-value volatiles in modern plantations.

Beyond genetic regulation, the spatial distribution of oil cells and the ultimate yield of these volatile compounds are highly responsive to developmental stages and environmental provenance. High-resolution anatomical studies during leaf ontogeny indicate that oil cell density and EO compositions fluctuate significantly, dictating optimal harvest windows to maximize specific terpenoid yields [23]. Furthermore, geographic profiling of various *C. officinarum* provenances highlights significant environmental influences on both leaf oil yield and chemotype distribution [46]. This necessitates targeted germplasm selection and geographically optimized agricultural practices to ensure the consistency and commercial viability of the EOs produced for the pharmaceutical and flavor industries.

Concurrently, the extraction methodologies utilized to harvest these thermolabile terpenes are undergoing a critical transition toward green chemistry and high-efficiency biorefining. While conventional steam distillation remains an industry standard, it frequently risks the thermal degradation of oxygenated monoterpenes and suffers from high energy consumption. Consequently, emerging techniques such as microwave-assisted hydrodistillation (MAHD) and pilot-plant neutral cellulase-assisted extraction have demonstrated superior efficacy in rupturing oil cells while maintaining the structural fidelity of the EOs [47,48]. Most notably, the application of deep eutectic solvents (DES), optimized through deep learning algorithms, has recently pioneered a circular extraction model. This advanced approach facilitates the simultaneous recovery of volatile EOs and structural biopolymers like lignin from *C. officinarum* leaves, drastically improving extraction efficiency while minimizing environmental impact [49].

### 3.2. Non-Volatile Secondary Metabolites

Although frequently overshadowed by the industrial focus on volatile terpenes, the non-volatile secondary metabolome of *C. officinarum* represents a vast, predominantly untapped reservoir of bioactive phytochemicals. This complex matrix comprises flavonoids, lignans, tannins, and alkaloids, which serve critical ecophysiological functions in defending the plant against oxidative stress, phytopathogens, and herbivores [50,51,52,53]. Recent advances in sub-tissue localization have mapped the intricate compartmentalization of these compounds, revealing a dense accumulation of polyphenols and condensed tannins in the epidermal tissues of leaves and stems, distinctly segregated from the secretory cells that harbor EOs [50]. This structural partitioning underscores the evolutionary strategy of *C. officinarum* to deploy broad-spectrum, non-volatile defenses alongside its volatile aromatic compounds.

The biosynthetic pathways responsible for these non-volatile metabolites are now being decoded through advanced functional genomics, revealing critical insights into their developmental regulation. Integrated transcriptomic and metabolomic analyses have recently traced the molecular mechanisms of flavonoid biosynthesis, illustrating how the plant dynamically partitions its carbon flux during leaf maturation [51]. By identifying the specific gene networks and transcription factors that coordinate the accumulation of these phenolics, researchers are uncovering the environmental and physiological triggers required to maximize non-volatile yields. This understanding is pivotal for optimizing agronomic practices aimed at harvesting camphor biomass specifically for its bioactive phenolic and alkaloid content, independent of its EO profile.

From an industrial and pharmacological perspective, the critical re-evaluation of these non-volatile fractions is catalyzing a shift toward zero-waste biorefining. Historically, the hydrolates effluent and spent leaf meal generated during EO hydrodistillation were discarded as biological waste. However, current literature increasingly emphasizes the substantial pharmacological and biorefinery value of these hydrolates. Because they retain hydrophilic bioactives during distillation, hydrolates offer a sustainable, upcycled source of polyphenols with robust antioxidant and antimicrobial properties [47,54]. Contemporary research has demonstrated that these residues retain high concentrations of potent antioxidant and anti-inflammatory flavonoids and lignans. Advanced recovery protocols have successfully isolated soluble and insoluble bioactive polyphenols from hydrolates and seed kernel residues, showcasing robust free-radical scavenging capabilities and notable acetylcholinesterase inhibitory activities [55]. By prioritizing the synergistic recovery of these non-volatile metabolites alongside EOs, future phytopharmaceutical applications can fully exploit the comprehensive therapeutic spectrum of *C. officinarum*, effectively transforming agro-industrial byproducts into high-value functional ingredients. To consolidate current research, a summary of non-volatile metabolites from various *C. officinarum* extracts is provided in Table 2.

### 3.3. Nutritional Composition of Seeds

Because *C. officinarum* is not a staple food crop, its nutritional significance within the botanical literature is primarily anchored in the exceptional fixed lipid profile of its seed kernels. *C. officinarum* seed kernel oil (CCSKO) has emerged as a premier, non-conventional botanical source of medium-chain triglycerides (MCTs). Comprehensive geographic and chemical profiling reveals that CCSKO contains an extraordinarily high concentration of MCFAs, frequently exceeding 95% of the total lipid fraction, which is overwhelmingly dominated by capric acid (C10:0, ~57–60%) and lauric acid (C12:0, ~35–40%) [56,57]. Unlike long-chain dietary fats, this unique triacylglycerol geometry allows MCTs to bypass the lymphatic system for rapid hepatic portal absorption and immediate oxidation, thereby mitigating adipose tissue deposition. This inherent metabolic advantage positions CCSKO as a highly competitive, sustainable alternative to commercial palm kernel and coconut oils.

The transition of CCSKO from a regional forestry byproduct to a globally recognized functional food ingredient has been facilitated by rigorous physiological and toxicological validation. Comprehensive sub-chronic toxicity testing, genotoxicity, and teratogenicity assessments have unequivocally confirmed the edible safety of the refined seed oil, overcoming traditional regulatory barriers and addressing historical hesitations surrounding camphor-derived products [58,59]. Furthermore, recent in vivo interventions in murine models demonstrate that dietary supplementation with CCSKO not only improves general lipid metabolism but also actively ameliorates oxidative stress and upregulates β3-adrenergic receptor expression in diet-induced obesity, highlighting its potential as a potent anti-obesity nutraceutical [60].

To fully exploit this functional lipid profile, food bioengineers are actively employing advanced lipid restructuring techniques to adapt CCSKO for broader commercial application. Through enzymatic interesterification, specific lipases are utilized to react CCSKO with long-chain triglycerides from other botanical stearins, such as fully hydrogenated palm oil or perilla seed oil. This biotransformation successfully synthesizes high-value, zero-trans structured lipids, yielding human milk fat analogs, specialized shortenings, and cocoa butter substitutes with superior rheological stability and optimized melting behavior [61,62]. Coupled with the recovery of high-quality proteins and prebiotic polysaccharides from the defatted kernel residue, the nutritional valorization of *C. officinarum* seeds represents a significant breakthrough in sustainable, plant-based food systems. The nutritional profile of *C. officinarum* seed kernel oil and its associated bioactive byproducts are presented in Table 3.

### 3.4. Applications in Animal Nutrition

The rapid expansion of the *C. officinarum* EO and seed lipid extraction industries has generated large volumes of agro-industrial byproducts, including spent leaf meals, degreased branches, and seed residues. Historically, these lignocellulosic wastes were considered recalcitrant due to high fiber content, astringent tannins, and residual anti-nutritional terpenoids, which reduced palatability and digestibility in livestock. However, driven by the push for a circular agricultural bioeconomy, recent advances in feed-processing methods have demonstrated that these residues can be upcycled into viable bulk fodder. Post-extraction treatments such as artificial drying, high-pressure pelleting, and anaerobic ensiling effectively degrade toxic volatile remnants while enhancing crude protein bioavailability. Processed degreased camphor branches, in particular, show improved in vitro ruminal fermentation, transforming potential ecotoxic waste into a sustainable, fiber-rich roughage that can help alleviate conventional feed shortages [64].

Beyond serving as a macronutrient substitute, residual *C. officinarum* biomass functions as a low-cost phytogenic feed additive (PFA). With the agricultural sector phasing out sub-therapeutic antibiotic growth promoters, trace secondary metabolites such as non-volatile polyphenols and residual monoterpenes in camphor spent meals offer immunomodulatory and antimicrobial effects. In vivo trials in monogastrics, such as Japanese quail, show that camphor supplementation modulates intestinal microbiota, enhances systemic cellular immunity, and improves zootechnical performance and hematological profiles [63]. These benefits extend to ruminants, with targeted dietary inclusion linked to higher live weights and optimized carcass traits in growing rams, reflecting positive nutrient partitioning [65]. These findings also validate ethnoveterinary practices, where farmers historically used dry camphor leaves in fodder to improve livestock health.

Regulatory advances further support the integration of camphor byproducts in commercial animal nutrition. Addressing concerns over dose-dependent neurotoxicity and hepatotoxicity, the European Food Safety Authority (EFSA) confirmed that camphor white oil, an EO fraction from *C. officinarum*, is a safe sensory and flavoring feed additive for all species at recommended levels [66]. The EFSA also found no environmental or human health risks associated with its use. Despite this, challenges remain in standardizing raw biomass, as chemotypic variation in *C. officinarum* leads to fluctuating levels of potentially toxic compounds. Future research should focus on chemotypic screening and optimizing inclusion thresholds to fully exploit the nutritional and prophylactic potential of camphor residues without compromising livestock welfare.

## 4. Multipurpose Uses

### 4.1. Ethnomedicine and Traditional Medicine

*C. officinarum* has served as a foundational botanical resource in global indigenous healing practices for centuries, most notably within Traditional Chinese Medicine (TCM), Ayurveda, and Unani systems [14,67]. Historically, diverse parts of the plant, including the bark, roots, and leaves, were formulated into decoctions and topical salves to treat a broad spectrum of ailments. In TCM and Ayurveda, crude camphor and EO fractions were prominently prescribed as analgesics and antispasmodics to alleviate rheumatism, bronchitis, and digestive lethargy [14,67]. Furthermore, historical texts record its utilization in mitigating infectious outbreaks; traditional Unani practitioners frequently employed camphor fumigation to combat epidemic fevers (*Humma-e-Wabai*), sudden collapse, and recurrent eruptive illnesses [15,68]. These ethnomedicinal practices intuitively leveraged the plant’s potent bioactive metabolites long before their precise chemical structures were elucidated, reflecting a deep empirical recognition of its broad-spectrum physiological efficacy.

Rather than relegating these applications to historical anecdotes, contemporary literature critically synthesizes and validates these traditional claims through modern toxicological and molecular paradigms. Recent safety assessments of ultra-high homeopathic dilutions of *C. officinarum* in preclinical models have confirmed a high safety margin with negligible organ toxicity, establishing vital thresholds for its continued therapeutic use [69]. Concurrently, advancements in network pharmacology are decoding the molecular rationale behind traditional holistic formulations. For instance, the traditional multi-herbal formulation Changyanning, which contains *C. officinarum* root extracts, has recently been shown to alleviate Crohn’s disease by specifically inhibiting GPX4-mediated ferroptosis, a sophisticated mechanism of regulated cell death [70]. Similarly, lignan-rich leaf extracts have been demonstrated to attenuate metabolic syndrome by modulating glycolipid metabolism and reshaping the gut microbiota in Type 2 diabetes models [52]. This paradigm shift from empirical folklore to mechanistic validation emphasizes that traditional ethnomedicine provides a highly accurate, pre-screened blueprint for modern phytopharmaceutical discovery.

### 4.2. Modern Pharmacological Activities

Modern pharmacological research has exponentially expanded the therapeutic profile of *C. officinarum*, moving beyond its classical reputation to uncover sophisticated, pathway-specific molecular interventions. The scientifically backed antimicrobial, anti-inflammatory, antioxidant, and antiviral properties of its EOs, particularly the linalool, cineole, and camphor chemotypes, are now well-documented. However, emerging research underscores the plant’s profound regulatory influence on complex cellular pathways rather than mere broad-spectrum toxicity. A critical frontier is its neuroprotective potential; recent studies demonstrate that *C. officinarum* EO can actively mitigate depression-like behaviors by regulating the Nrf2/HO-1 signaling pathway and suppressing neuroinflammatory cytokines [71]. This provides a mechanistic basis for its psychopharmacological applications, pivoting its modern use toward neuroprotective drug development.

In addition to neuroprotection, groundbreaking insights have emerged regarding the plant’s efficacy in treating oncological disorders, shifting attention toward specific bioactive isolates and non-volatile fractions. In the realm of oncology, (+)-2-bornanone, a key volatile constituent, has been shown to exert potent anti-tumor effects in breast cancer by directly modulating NOX4, uncovering a previously unknown oxidative stress-mediated apoptotic pathway in malignant cells [44]. Despite these profound in vitro and in vivo successes, a critical bottleneck remains in clinical translation. The high volatility, poor aqueous solubility, and potential dose-dependent toxicity of camphor-rich fractions require the concurrent development of advanced, targeted drug delivery systems. This synthesis highlights a critical evolution in the literature: *C. officinarum* is increasingly recognized not just as a source of topical counter-irritants but as a reservoir of highly purified, stereospecific bioactives capable of targeted interventions in chronic and systemic diseases when paired with next-generation encapsulation technologies. The validated pharmacological activities and targeted molecular mechanisms of these constituents are synthesized in Table 4.

However, a critical appraisal of these pharmacological findings reveals a significant gap between in vitro success and clinical utility. While these pathway-specific interventions are promising, most studies rely on isolated cell lines or murine models, which may not accurately reflect human metabolic responses to volatile terpenoids. Furthermore, the lack of standardized extraction protocols across different studies makes it difficult to compare the efficacy of extracts from different chemotypes, highlighting a pressing need for more rigorous, human-centered clinical trials and unified methodological standards.

### 4.3. Agricultural and Ecological Uses

The application of *C. officinarum* in agriculture has transcended traditional contact repellency, evolving into the targeted use of botanical biopesticides, larvicides, and resistance-inducing agents. Secondary metabolites derived from the tree exhibit significant toxicity against destructive agricultural pests and disease vectors, offering a biodegradable alternative to synthetic agrochemicals. However, a critical analysis of plant–insect chemical ecology reveals a complex evolutionary arms race rather than simple unilateral toxicity. While camphor is highly toxic to generalist pests, specialized monophagous insects, such as the camphor tree moth (*Orthaga achatina*), have evolved specific odorant receptors that recognize camphor, turning a potent plant defense mechanism into a host-location cue [72]. Beyond direct insecticidal activity, specific chemotypes act as potent biological elicitors; for instance, linalool emitted from the plant induces systemic resistance against the Tobacco Mosaic Virus (TMV) in neighboring crops [40], illustrating a sophisticated ecological utility in integrated pest management.

Ecologically, the camphor tree has emerged as a premier candidate for phytoremediation, specifically in urban and industrially compromised environments burdened by heavy metal contamination. The plant possesses a remarkable intrinsic tolerance to toxic elements, actively cleaning soils of heavy metals like copper and cadmium. Recent investigations into root dynamics reveal a highly adaptive spatial heterogeneity, where roots structurally and biochemically alter their antioxidant capacity depending on their proximity to excessive copper stress [33]. Furthermore, researchers have discovered that the application of exogenous elicitors, such as melatonin, dramatically enhances the plant’s capacity to absorb and detoxify cadmium-contaminated soils [73]. These insights suggest that genetically or hormonally optimized *C. officinarum* cultivars could serve as highly efficient, scalable biocovers for the targeted reclamation of post-industrial brownfields and the biological control of degraded ecosystems.

### 4.4. Industrial and Commercial Applications

The industrial valorization of *C. officinarum* is undergoing a radical transformation, pivoting from traditional bulk extraction to high-tech materials science and sustainable biotechnology. While the high-yield extraction of linalool and natural D-borneol remains a commercial staple for the global perfumery and cosmetics markets, modern innovations are increasingly utilizing camphor derivatives in active food preservation. For instance, the food industry is actively integrating *C. officinarum* phytochemicals into sustainable packaging by embedding anthocyanins extracted from the tree’s fruit peels into sodium carboxymethyl cellulose-gum Arabic biopolymer films. These smart, natural food preservatives create novel, low-temperature dehydration and antimicrobial packaging systems for meat, perfectly merging botanical phytochemistry with sustainable food engineering [74].

Simultaneously, the commercial applications of *C. officinarum* are expanding into novel biochemical domains, ensuring the total utilization of the plant’s biomass in a circular bioeconomy. CCSKO, traditionally discarded, is now subjected to enzymatic interesterification to produce highly structured lipids with improved physicochemical and rheological properties, making it an ideal ingredient for functional foods and detergents [62]. In the realm of advanced material sciences, the unique microstructural architecture of *C. officinarum* bark has been combined with the electrostatic self-assembly of MXene and chitosan to engineer smart cotton fabrics that offer highly sensitive micro-breathing monitoring alongside rapid photothermal antibacterial properties [75]. Conversely, a critical re-evaluation of traditional commercial applications is also underway. While camphor wood has been historically prized for crafting insect-repellent museum storage cabinets, modern analytical chemistry has identified continuous VOC emissions from the wood as a potential degradation risk to sensitive, long-term heritage collections [76]. This critical reappraisal highlights the necessity of balancing traditional material uses with modern conservation science, ensuring the safe and optimized industrial application of *C. officinarum*.

## 5. Biotechnology and Molecular Biology

### 5.1. Plant Tissue Culture and Micropropagation

The conventional agricultural expansion of *C. officinarum* is severely constrained by its reproductive biology, which is characterized by poor seed viability, slow germination rates, and extreme genetic heterozygosity. To overcome these agronomic bottlenecks, in vitro micropropagation has become a foundational tool for the rapid clonal multiplication of elite, high-yielding chemotypes. Early optimization efforts established that successful micropropagation from nodal segments and shoot tips is highly genotype-dependent, requiring precise formulations of woody plant medium supplemented with cytokinins like benzyladenine (BA) to induce shoot proliferation [77,78,79]. Importantly, molecular validation using random amplified polymorphic DNA analysis confirms that these in vitro-generated plantlets maintain strict genetic fidelity with the mother plant, ensuring that the distinctive EO profiles of selected chemotypes are preserved throughout the commercial supply chain [80].

Beyond basic clonal multiplication, contemporary research has pivoted toward advanced morphogenic systems, particularly somatic embryogenesis and protoplast isolation. The establishment of cyclic secondary somatic embryogenesis represents a critical biotechnological leap, as it circumvents the limitations of explant scarcity and provides an indefinitely scalable, high-efficiency regeneration pathway [81]. Simultaneously, the successful isolation and plantlet regeneration of *C. camphora* protoplasts from embryogenic suspension cultures provide a powerful cellular platform for somatic hybridization, opening the door for future CRISPR/Cas9 gene-editing applications [82]. These advanced morphogenic pathways do not merely serve to scale vegetative propagation; they supply an indispensable, totipotent cellular reservoir for functional genomics and the engineering of targeted physiological traits, such as disease resistance or tailored EO profiles.

Despite these successes, in vitro recalcitrance during the rooting phase remains a historic challenge for woody species like *C. camphora*. However, recent multidisciplinary studies have transitioned the field from empirical media trials to precision, transcriptomically guided tissue culture. By integrating physiological monitoring with RNA sequencing during adventitious root formation, researchers have decoded the intricate molecular crosstalk underlying organogenesis, illustrating how phytohormones regulate a cascade of downstream genes responsible for mitigating oxidative stress [83]. Furthermore, researchers are leveraging callus and cell suspension cultures not merely for plant regeneration but as standalone in vitro biofactories. The application of elicitors like methyl jasmonate to suspension cultures has been shown to drastically upregulate the biosynthesis of high-value compounds such as ionones, circumventing the need for the destructive harvesting of mature trees while yielding potent, antioxidant-rich cellular biomass [53,84].

### 5.2. Genomics, Transcriptomics, and Multi-Omics

The transition from classical phytochemistry to molecular systems biology in *C. officinarum* has been catalyzed by the recent assembly of high-quality, chromosome-level reference genomes. These structural blueprints have illuminated the profound evolutionary history of the Lauraceae family, identifying specific WGD events that drove the massive expansion and neofunctionalization of gene clusters responsible for terpenoid and triglyceride biosynthesis [20,85]. By serving as an anchoring reference, this genomic architecture has enabled extensive whole-genome resequencing and the systematic identification of critical transcription factor families, such as the MYB gene superfamily, which regulates secondary metabolism and stress responses [86]. These genome-wide maps have revealed the underlying loci dictating the tree’s robust ecological adaptability, providing an invaluable genomic repository for future marker-assisted selective breeding.

With the genome resolved, RNA sequencing has become an indispensable tool for mapping the dynamic spatiotemporal regulation of the plant’s vast secondary metabolism. Early transcriptomic profiles comparing distinct chemical phenotypes (e.g., linalool, borneol, and citral chemotypes) successfully isolated the differentially expressed genes within the mevalonate and methylerythritol phosphate pathways [45]. Crucially, transcriptomics has demonstrated that chemotypic variation is driven heavily by the extreme transcriptional plasticity of late-stage synthase enzymes. Furthermore, RNA sequencing is increasingly utilized to decipher the plant’s physiological resilience. Recent studies on cold stress responses and foliar color transitions have identified key gene networks that simultaneously govern ROS scavenging, cryoprotectant synthesis, and anthocyanin metabolite formation, linking metabolic defense mechanisms with environmental adaptation [87,88].

The current frontier of *C. officinarum* systems biology lies in the integration of multi-omics, coupling high-resolution transcriptomics with mass spectrometry-based metabolomics. Rather than viewing genes in isolation, researchers are now mapping comprehensive metabolic fluxes across varying developmental stages and tissues. For example, recent dual-omics investigations into stem development have uncovered the competitive carbon allocation between secondary cell wall deposition (lignification) and terpenoid biosynthesis, offering a holistic view of how the tree balances structural growth with chemical defense [26]. Similarly, multi-omics profiling of the highly prized citral chemotype has delineated the exact gene–metabolite networks driving citral hyperaccumulation in developing leaves [22]. This systems-level insight is transformative, transitioning botanical research from descriptive profiling to predictive metabolic modeling, which is essential for the targeted bioengineering of elite cultivars.

### 5.3. Biosynthetic Pathways and Metabolic Engineering

The immense commercial value of *C. officinarum* EOs is strictly governed by the evolutionary divergence of the TPS gene superfamily, which acts as the primary enzymatic gatekeeper of the plant’s chemodiversity. Recent functional genomic characterizations have identified dozens of unique TPS genes, mapping the precise amino acid motifs that direct universal prenyl diphosphate precursors into specific monoterpene and sesquiterpene scaffolds [35]. Landmark breakthroughs include the isolation of a highly specialized deduced geraniol synthase that acts as the critical bottleneck for citral production [21], as well as the evolutionary reconstruction of a sesquiterpene synthase (santalene synthase) that originated from a monoterpene-synthesizing ancestor [89]. The systematic identification of these chemical forms clarifies how subtle, localized genetic variations dictate the drastically different volatile signatures defining the species’ numerous industrial chemotypes.

While identifying structural TPS genes provides the foundation, contemporary research increasingly focuses on the complex regulatory networks and late-stage oxidative modifications that finalize the EO’s profile. Advanced studies have revealed that terpenoid accumulation is heavily regulated by transcription factors, such as the bHLH family, which act as master switches triggering volatile and anthocyanin biosynthesis in response to mechanical damage and abiotic stress [43]. Furthermore, the molecular cloning of a high-efficiency (+)-borneol dehydrogenase has mapped the terminal oxidation step connecting the borneol and camphor pathways, illustrating that final EO yields are dependent on continuous metabolic flux and interconversion [41]. The comprehensive mining of these candidate genes elucidates the camphor biosynthesis pathway, offering a precise molecular roadmap for understanding chemotype differentiation [90].

Synthesizing this profound understanding of *C. officinarum*’s enzymatic machinery, modern biotechnology is shifting focus toward microbial metabolic engineering to sustainably bypass the limitations of traditional agriculture. In a monumental leap for synthetic biology, researchers have successfully transplanted the entire borneol and camphor degradation and synthesis pathways into engineered *Pseudomonas* hosts [42]. By artificially optimizing precursor pools and fine-tuning the expression of these heterologous plant genes, bioengineers have achieved the scalable biomanufacturing of optically pure bicyclic monoterpenes. Furthermore, utilizing systematic identification and chemical expression forms of key TPS in heterologous systems accelerates the commercial production of rare terpene variants [91]. This paradigm shift toward microbial biofactories represents the ultimate application of mapping the camphor tree’s genome, fundamentally disrupting the reliance on wild forest harvesting.

### 5.4. Advanced Extraction Technologies

Industrial extraction of *C. officinarum* EOs has traditionally relied on steam and hydrodistillation, but these methods are thermodynamically inefficient and can degrade thermally sensitive monoterpenes. To overcome these limitations, supercritical fluid extraction (SFE) with carbon dioxide has been widely adopted as a greener alternative. Because it operates at adjustable high pressures and near-ambient temperatures, SFE protects the plant matrix from oxygen and excessive heat while selectively dissolving non-polar terpenoids [92]. As a result, it improves EO recovery, preserves trace aromatic compounds that are often lost during heating, and maintains the stereochemical integrity and natural aroma of the volatiles without leaving toxic solvent residues [93].

Microwave-assisted extraction (MAE) has also greatly improved EO recovery by using electromagnetic radiation for rapid volumetric intracellular heating. This causes rapid rupture of the glandular oil cells, reducing extraction time from hours to minutes while increasing yield. Recent response surface methodology optimization MAHD for camphor leaves showed that this approach can recover high-quality volatile oils while also enabling the extraction of valuable water-soluble polyphenols from the remaining aqueous phase [47,94]. In addition, MAE supports targeted recovery of proanthocyanidins and other antioxidants from the leaves, supporting an efficient biorefinery strategy that enhances the therapeutic value of the final extract [54].

Even when advanced extraction produces highly purified EOs, their volatility, poor water solubility, and susceptibility to oxidation still limit direct biomedical and industrial use. To address this, researchers are increasingly applying nanotechnology to formulate these oils into stable nanoemulsions. High-shear homogenization reduces droplet size to the nanoscale, greatly increasing the surface-area-to-volume ratio of the active compounds. These nanoemulsions show improved bioavailability and stronger targeted activity. For example, Ho Wood nanoemulsions combined with UV-C LED irradiation act as effective biopreservatives against *Staphylococcus aureus* in plant-based foods [95], while chitosan-loaded nanoemulsions help prevent postharvest aflatoxin contamination [96] and show strong eco-friendly acaricidal activity against cattle ticks [97]. A comparative summary of these advanced extraction technologies and their biotechnological impacts is provided in Table 5.

## 6. Toxicity, Safety, and Ecological Challenges

### 6.1. Human Toxicity and Safe Dosages

While *C. officinarum* offers extensive pharmacological utility, its primary bioactive constituent, the bicyclic monoterpene ketone camphor, presents significant toxicological risks characterized by a notoriously narrow therapeutic index. Acute camphor intoxication is predominantly marked by rapid-onset neurotoxicity; owing to its high lipid solubility, camphor swiftly crosses the blood–brain barrier, precipitating central nervous system (CNS) overstimulation that manifests as delirium, lethargy, and refractory generalized seizures [98,99]. Beyond neurotoxicity, contemporary toxicological syntheses emphasize the severe hepatotoxic potential of excessive camphor ingestion. The liver serves as the primary metabolic clearinghouse for monoterpenes; acute or chronic overdosing heavily burdens hepatic hydroxylation pathways, leading to the accumulation of reactive intermediates that induce massive oxidative stress, lipid peroxidation, and hepatocyte necrosis [100]. Furthermore, emerging in vivo models utilizing zebrafish embryos have demonstrated that sub-lethal camphor exposure triggers systemic oxidative stress, structural developmental alterations, and profound cardiotoxicity, underscoring the systemic vulnerabilities associated with this compound [101].

The risk profile of camphor is drastically elevated in vulnerable demographic groups, making its use strictly contraindicated in pediatric care and during pregnancy. Pediatric populations are highly susceptible to camphor-induced neurotoxicity due to an absence of fully mature hepatic glucuronidation enzymes required for efficient terpene metabolism. Clinical case reports repeatedly highlight that accidental ingestion or excessive dermal application of camphor-containing traditional remedies and over-the-counter (OTC) rubefacients can trigger severe symptomatic convulsions in toddlers, frequently necessitating emergency intervention [102,103]. Consequently, maternal and fetal risks are equally severe; camphor is widely recognized as an embryo–fetotoxic agent that exhibits maternal reproductive toxicity, potential teratogenicity, and hormone modulation, strictly prohibiting its use by pregnant and lactating women [104].

To mitigate these toxicological hazards, modern regulatory agencies, such as the U.S. Food and Drug Administration (FDA), universally restrict the camphor concentration in topical OTC products to a maximum of 11%, effectively curbing the incidence of fatal out-of-hospital poisonings [98]. However, a critical synthesis of recent research demonstrates that toxicity is highly dependent on the plant fraction utilized, highlighting new avenues for safe consumption. For instance, recent evaluations of CCSKO, a fraction rich in MCTs but depleted of toxic foliar monoterpenes, demonstrated a complete absence of sub-chronic toxicity, genotoxicity, and teratogenicity, confirming its safety as a novel dietary supplement [58,59]. Additionally, rigorous assessments of ethanol-potentized, ultra-high dilutions (homeopathic preparations) of *C. officinarum* confirmed a lack of acute or subacute oral toxicity in murine models [69]. This divergence necessitates a paradigm shift in regulatory risk assessments, distinguishing the severe systemic toxicity of concentrated camphor from the benign nature of the plant’s lipid-rich seed extracts and highly diluted formulations.

### 6.2. Ecological Invasiveness

Although native and highly valued in East Asia, *C. officinarum* presents a complex ecological paradox: originally disseminated globally in the late 19th and early 20th centuries for ornamental landscaping and timber, it has aggressively transitioned into a formidable invasive weed in numerous subtropical and tropical non-native ecosystems. In regions such as Florida, Hawaii, and eastern Australia, the tree’s robust biological traits, including rapid vegetative growth, broad climatic tolerance, and prolific seed production, have enabled it to readily escape cultivation [10,105]. Once established in disturbed forests and riparian zones, *C. officinarum* rapidly forms dense, monospecific canopies that obstruct light penetration, outcompeting native understory flora and drastically altering local forest succession. This competitive exclusion poses a cascading threat to local fauna, displacing indigenous avian populations that rely on endemic plant hosts while actively facilitating invasions by other exotic species, such as harboring the invasive Argentine ant (*Linepithema humile*) in honeydew-producing insect networks [106,107].

A primary mechanism driving the ecological dominance of *C. officinarum* is its potent allelopathic capability, which actively engineers the soil environment to favor its own propagation. The tree continuously leaches a complex matrix of volatile secondary metabolites, predominantly camphor, linalool, and eucalyptol, into the soil matrix via root exudation and the decomposition of its abundant leaf litter [108]. These allelochemicals induce severe physiological damage to surrounding native species by promoting hydrogen peroxide and malondialdehyde accumulation, disrupting antioxidant enzyme systems, and inhibiting vital osmoregulation, ultimately arresting seed germination and vegetative growth [109]. Evolutionary ecology studies suggest that invasive populations of *C. officinarum* often exhibit higher chemical diversity and stronger chemical defenses compared to their native counterparts, deterring local herbivores and accelerating invasion dynamics [110]. Interestingly, a contemporary dual-use paradox has emerged in weed science: the potent allelopathic extracts of *C. officinarum* can significantly inhibit the vegetative reproduction of other aggressive global weeds, such as *Alternanthera philoxeroides*, presenting a novel opportunity to harness its phytotoxicity as a natural bioherbicide [109]. The comprehensive toxicological risks, safety boundaries, and ecological impacts discussed in this section are consolidated in Table 6.

The ecological management of *C. officinarum* requires a multifaceted, landscape-level strategy, as traditional mechanical removal often exacerbates the problem by triggering vigorous basal coppicing and root suckering. Current ecological management paradigms rely heavily on targeted chemical control (e.g., cut-stump or basal bark herbicide application) integrated with the systematic restoration of competitive, allelopathy-resistant native canopy species [10]. Furthermore, anticipating future invasions requires advanced predictive modeling; current ecological risk assessments utilizing MaxEnt-based habitat projections indicate that anthropogenic climate change will likely expand the globally suitable environments for *C. officinarum*, exacerbating its potential spread into newly vulnerable latitudes [11,111]. This underscores the critical need for continuous urban monitoring, as urban trees often serve as permanent reservoirs for both expanding weed populations and insect pests, and for proactive international quarantine protocols to mitigate further ecological disruption [112].

**Table 6:** Toxicological risks, safety boundaries, and ecological impacts.

Hazard Category	Specific Agent/Toxic Mechanism	Vulnerable Targets and Biological Consequences	Management Strategies/Safety Thresholds	References
**Acute human toxicity**	Camphor-induced CNS overstimulation and hepatic hydroxylation burden	**Targets:** Pediatric populations (immature hepatic enzymes); General populace via overdose. **Consequences:** Seizures, delirium, hepatocyte necrosis.	**FDA Regulation:** Restricts OTC topical formulations to a maximum of 11% camphor.	[98,100,103]
**Developmental toxicity**	Embryo–fetotoxicity, Cardiotoxicity	**Targets:** Pregnant and lactating women; developing embryos. **Consequences:** Triggers systemic oxidative stress and structural alterations.	Strictly contraindicated for maternal and pediatric use.	[101,104]
**Ecological invasiveness**	Rapid vegetative growth, shade-tolerant dense canopies	**Targets:** Subtropical non-native ecosystems (e.g., Florida, eastern Australia). **Consequences:** Displaces endemic flora/fauna; facilitates secondary invasions (Argentine ant).	Managed via chemical control integrated with native canopy restoration and urban monitoring.	[10,107,112]
**Allelopathy**	Root exudation/leaf litter leaching (camphor, linalool, eucalyptol)	**Targets:** Surrounding native plant seeds and understory. **Consequences:** Arrests seed germination via hydrogen peroxide accumulation.	**Dual-use opportunity:** Harnessing phytotoxicity to develop natural bioherbicides against other global weeds.	[108,109]
**Safe usage fractions**	CCSKO and Ultra-high homeopathic dilutions	**Targets:** Human consumers. **Consequences:** Negligible organ toxicity.	Proven edible safety for CCSKO (zero sub-chronic toxicity, genotoxicity, or teratogenicity).	[58,59,69]

Despite the identification of these allelopathic mechanisms, the practical application of *C. officinarum* as a bioherbicide remains fraught with complexity. The high volatility of the active compounds makes maintaining effective soil concentrations difficult in field conditions, and there is a critical risk that utilizing invasive populations for herbicide production could inadvertently incentivize the maintenance of these trees in non-native regions, creating a conflict between economic gain and ecological restoration goals.

## 7. Conclusions and Future Perspectives

### 7.1. Summary of Key Findings

The study and commercial use of *C. officinarum* have shifted from traditional empirical practices to modern, genomic-based biotechnology. Taxonomically, genetic analyses have resolved historical inaccuracies, reclassifying the species into the distinct *Camphora* lineage. Biologically, the plant is known for its high chemical diversity and physiological adaptability. Recent genome assemblies show that this diversity is linked to ancient gene duplications and the expansion of the TPS gene family, which controls the production of valuable EO chemotypes.

In terms of phytochemistry, the industry is moving toward more sustainable, zero-waste processing. Green extraction methods, such as SFE and MAE, are now used alongside the recovery of non-volatile polyphenols and biomass for animal feed. Additionally, CCSKO has been identified as a safe, functional dietary source of MCTs. Pharmacologically, molecular studies support many of the plant’s traditional medical uses, revealing specific neuroprotective and anti-tumor properties. At the same time, biotechnological methods like tissue culture and microbial cell factories offer new ways to produce these compounds without relying on wild tree harvesting. However, challenges remain: the tree is highly invasive outside its native range, and the narrow safety margin of camphor requires strict toxicological oversight.

### 7.2. Current Research Gaps

Despite recent progress, several barriers limit the commercial and clinical application of *C. officinarum*. First, while animal and cell models show promising anti-inflammatory, neuroprotective, and anti-tumor effects from specific derivatives (e.g., (+)-2-bornanone), well-designed human clinical trials are currently lacking. Clinical use is hindered by the high volatility, low water solubility, and potential neurotoxicity of terpene-rich extracts.

Second, although CCSKO has passed safety and toxicity tests, long-term human dietary studies are needed to confirm its benefits for weight management and lipid regulation. The large-scale production of structured lipid products from CCSKO also requires further sensory and physical testing.

In food science and technology, several specific gaps remain unresolved. Although *C. officinarum* seed kernel oil shows promise as a medium-chain triglyceride-rich functional ingredient, more work is needed to evaluate its flavor stability, oxidative behavior during storage, consumer acceptability, and performance in real food systems such as bakery fats, dairy analogs, infant-formula fat substitutes, and nutraceutical emulsions. In addition, the application of *C. officinarum*-derived essential oils, polyphenols, and anthocyanins in active packaging and food preservation requires stronger evidence on migration behavior, sensory impact, antimicrobial efficacy under commercial processing conditions, and regulatory safety. Future food technology studies should also standardize extraction, encapsulation, and formulation strategies to ensure batch-to-batch consistency and safe use of different chemotypes.

Third, while engineering microbes (like *Pseudomonas*) to produce camphor and borneol is a major step forward, this technology is currently restricted to the laboratory. Scaling up these microbial systems to achieve commercially viable production remains a significant biochemical engineering challenge.

Finally, using leftover camphor leaf meal and branches in animal feed is complicated by the plant’s highly variable chemical makeup. Without standardized screening protocols to measure leftover toxic terpenes, it is difficult to establish universal safe dietary limits for livestock.

### 7.3. Future Directions for Sustainable Use

To maximize the benefits of *C. officinarum* while ensuring ecological sustainability, future research should focus on a few strategic areas.

First, because climate change is expected to alter the habitats of valuable EO chemotypes, researchers should use newly assembled reference genomes, marker-assisted selection, and gene-editing tools like CRISPR/Cas9 to breed resilient, high-yielding cultivars that can survive shifting environmental stresses.

Second, processing methods should move toward fully integrated biorefineries. By using AI-optimized green extraction techniques, such as DES, industries could extract volatile terpenes, polyphenols, seed lipids, and biopolymers from the same batch of plant material, effectively eliminating agricultural waste.

Third, the pharmaceutical and food industries should invest heavily in nanotechnology. Encapsulating sensitive terpenes in nanoemulsions, liposomes, or biopolymer matrices can improve their stability and absorption while lowering the risk of systemic toxicity.

Lastly, in regions where *C. officinarum* is invasive, management should shift from simple eradication to “harvest-for-control” approaches. Extracting useful biopesticides and materials from invasive populations can help fund local ecological restoration. At the same time, large, old native trees in East Asia must be strictly protected as irreplaceable sources of genetic diversity.

In conclusion, *C. officinarum* is a multipurpose tree species with considerable value for phytochemistry, functional foods, pharmaceuticals, agriculture, and biotechnology, but its future use must be guided by a balanced understanding of both its benefits and risks. Current evidence highlights major opportunities in chemotype-based germplasm selection, green extraction, integrated biorefining, seed-oil valorization, nano-enabled delivery systems, and microbial production of valuable terpenoids. At the same time, important knowledge gaps remain in clinical validation, long-term dietary safety, chemotype standardization, large-scale metabolic engineering, and the safe use of residual biomass. In introduced regions where the species behaves invasively, exploitative control strategies such as harvesting invasive biomass for essential oils, biopesticides, or industrial materials may help offset management costs, provided that such use does not encourage further planting or persistence of invasive populations. Therefore, future research and management should integrate sustainable utilization, conservation of native genetic resources, and strict ecological control in non-native habitats.

## Figures and Tables

**Table 1:** Major essential oil chemotypes, key biosynthetic drivers, and applications.

Chemotype	Dominant Volatile Compounds	Key Biosynthetic Genes/Enzymes	Industrial and Ecological Applications	References
**Citral**	Citral	Geraniol synthase (*CoGES*)	Advanced flavorings, high-value pharmaceuticals; precision biomanufacturing.	[21,22,23]
**Linalool**	Linalool	TPS gene superfamily	High-end perfumery, cosmetics; acts as a biological elicitor, inducing systemic resistance against TMV in crops	[35,40]
**Borneol**	Natural D-borneol	(+)-borneol dehydrogenase	Advanced flavorings, pharmaceuticals; target for microbial biomanufacturing	[41,42]
**Camphor**	Camphor, (+)-2-bornanone	Borneol degradation pathways, bHLH transcription factors	Traditional analgesics, biopesticides; yields high-value anti-tumor isolates	[35,43,44]
**Cineole**	1,8-cineole (Eucalyptol)	Late-stage synthase enzymes	Antimicrobial applications; acts physiologically in the plant to scavenge ROS	[30,45]

**Table 2:** Summary of non-volatile metabolites of *C. officinarum*.

Extract Source	Metabolite Classes	Extraction Method	Identification Technique	Yield	References
**Leaves and stems**	Polyphenols, condensed tannins	Solvent micro-extraction, histochemical sectioning	Light/fluorescence microscopy, LC-MS	High density in epidermal tissues	[50]
**Developing leaves**	Flavonoids	Solvent extraction (methanol/water)	UPLC-ESI-MS/MS	Variable (dependent on leaf developmental stage)	[51]
**Seed kernel residue**	Soluble and insoluble polyphenols	Ultrasonic-assisted alkaline/acid extraction	HPLC-DAD, in vitro assays	Concentration varies by fraction	[55]
**Hydrolates (extract fluid)**	Water-soluble polyphenols	Microwave-assisted hydro-distillation (MAHD)	Spectrophotometry, HPLC	Up to ~12.5 mg GAE/g	[47]
**Leaves**	Lignans, flavonoids	Ethanol extraction, macroporous resin purification	UPLC-Q-TOF-MS	High recovery of bioactive components	[52]
**Leaves**	Proanthocyanidins	Microwave-assisted extraction (MAE)	UV-Vis spectrophotometry, HPLC-MS	Optimized up to ~11.23 mg/g	[54]

**Table 3:** Nutritional profile and bioactive non-volatile byproducts.

Plant Fraction	Major Bioactive/Nutritional Constituents	Physiological Benefits and Commercial Uses	References
**CCSKO**	MCFAs: Capric acid (C10:0, ~57–60%), Lauric acid (C12:0, ~35–40%)	Bypasses lymphatic system for rapid hepatic portal absorption; upregulates β3-adrenergic receptors as an anti-obesity nutraceutical.	[56,57,60]
**Structured lipids (Processed CCSKO)**	Zero-trans structured lipids	Synthesized via enzymatic interesterification for human milk fat analogs, specialized shortenings, and cocoa butter substitutes.	[61,62]
**Aqueous effluent and spent leaf meal**	Flavonoids, Lignans, Condensed Tannins	Exhibits robust free-radical scavenging and acetylcholinesterase inhibition; attenuates metabolic syndrome in T2DM.	[52,55]
**Degreased branches/residual biomass**	Trace monoterpenes, crude protein, prebiotic polysaccharides	Upcycled as phytogenic feed additives (PFAs); improves in vitro ruminal fermentation and intestinal microbiota in livestock.	[63,64]

**Table 4:** Validated pharmacological activities and targeted molecular mechanisms.

Pharmacological Target	Active Constituent/Extract	Observed Pharmacological Effect and Mechanism of Action	References
**Depression (Neurology)**	EO (Mixed monoterpenes)	Mitigates depression-like behaviors by regulating the Nrf2/HO-1 signaling pathway and suppressing neuroinflammatory cytokines.	[71]
**Breast cancer (Oncology)**	(+)-2-bornanone (Volatile isolate)	Exerts potent anti-tumor effects by directly modulating NOX4, triggering oxidative stress-mediated apoptosis in malignant cells.	[44]
**Crohn’s disease (Gastroenterology)**	Root extract (in *Changyanning* formulation)	Alleviates intestinal inflammation by specifically inhibiting GPX4-mediated ferroptosis (regulated cell death).	[70]
**Metabolic syndrome (Endocrinology)**	Lignan-rich leaf extract	Attenuates metabolic syndrome by modulating glycolipid metabolism and actively reshaping gut microbiota in Type 2 diabetes.	[52]

**Table 5:** Advanced extraction technologies and biotechnological innovations.

Technology/Method	Application Area	Primary Advantages and Commercial Impact	References
**SFE**	Green extraction	Utilizes pressurized CO_2_ at near-ambient temperatures; prevents thermal degradation of terpenoids and leaves zero toxic solvent residues.	[92,93]
**MAE**	Biomass biorefining	Electromagnetic rupture of cells reduces extraction to minutes; allows simultaneous recovery of volatile EOs and water-soluble polyphenols.	[47,54]
**DES**	Circular bioeconomy	AI-optimized solvents facilitate the simultaneous recovery of volatile EOs and structural biopolymers (e.g., lignin) from leaves.	[49]
**Microbial metabolic engineering**	Synthetic biology	Transplantation of borneol/camphor pathways into engineered *Pseudomonas* hosts allows scalable biomanufacturing without wild forest harvesting.	[42]
**Nanotechnology (Nanoemulsions)**	Advanced delivery systems	High-shear homogenization exponentially enhances terpene bioavailability; proven effective as a targeted bio-preservative and eco-friendly acaricide.	[95,97]

## Data Availability

No new data were created or analyzed in this study. Data sharing is not applicable to this article.

## Abbreviations

VOCs	Volatile organic compounds
WGD	Whole-genome duplication
TPS	Terpene synthase
MaxEnt	Maximum entropy
ROS	Reactive oxygen species
EOs	Essential oils
MAHD	Microwave-assisted hydrodistillation
DES	Deep eutectic solvents
CCSKO	*C. officinarum* seed kernel oil
MCTs	Medium-chain triglycerides
MCFAs	Medium-chain fatty acids
PFAs	Phytogenic feed additives
TMV	Tobacco mosaic virus
SFE	Supercritical fluid extraction
MAE	Microwave-assisted extraction
CNS	Central nervous system
OTC	Over-the-counter
FDA	Food and Drug Administration
